# Contributions of the hair sheep breed Santa Ines as a maintenance host for *Rhipicephalus* (*Boophilus*) *microplus* (Acari: Ixodidae) in Brazil

**DOI:** 10.1186/s13071-014-0515-5

**Published:** 2014-11-18

**Authors:** Marcos Valério Garcia, Renato Andreotti, Fernando Alvarenga Reis, André de Abreu Rangel Aguirre, Jacqueline Cavalcante Barros, Jaqueline Matias, Wilson Werner Koller

**Affiliations:** Molecular Biology Laboratory, Embrapa Beef Cattle, Campo Grande, MS Brazil; Embrapa Goats and Sheeps, Sobral, CE Brazil; Graduate Program of Infectious and Parasitic Diseases, School of Medicine, Universidade Federal de Mato Grosso do Sul (UFMS), Campo Grande, MS Brazil

**Keywords:** *Rhipicephalus microplus*, Santa Ines hair sheep, Pastures, Biological cycle, Brazil

## Abstract

**Background:**

Hair sheep breeds are a new, cost-effective option for the diversification of livestock in the Midwest region of Brazil. They are grazed extensively with cattle as well as in isolation in small areas. Hair sheep breeds are vulnerable to infestation by parasites such as the cattle tick, *Rhipicephalus microplus*, which causes various types of damage and can transmit diseases.

**Methods:**

In this study, Santa Inês hair sheep were naturally infested in an area contaminated by infested cattle and then monitored to determine the ability of these animals to maintain the local tick population in the absence of cattle. After engorged tick females of each generation fell off, the animals were placed in another pasture and were returned only after larvae reappeared in the original pasture.

**Results:**

Tick counts were performed every ten days for three generations of sheep, and average infestations per animal of 34, 12 and 4 ticks were observed for each successive generation. These numbers suggest the acquisition of resistance; however, additional studies are needed to ensure resistance is achieved. The average length of the parasitic phase for each generation of ticks was 25 days.

**Conclusion:**

We concluded that this hair sheep breed, even if kept separate from cattle, is able to maintain tick populations for at least three generations, although a gradual decrease in the population levels of *R. microplus* over three generations was observed. We also detected two positive cases of *Anaplasma* spp. Therefore, it appears that the Santa Inês hair sheep breed contributes to the circulation of this bacterium among other ruminants.

## Background

The Santa Inês hair sheep breed is originally from the northeastern region of Brazil, and is popular in the Brazilian Midwest for breeding to produce lambs for meat production because the weather in this region is favorable for this industry [1]. In areas with more extensive livestock production in the savanna, grazing of sheep with beef cattle is common [2], but may create health complications for both species [3]. The cattle tick *Rhipicephalus* (*Boophilus*) *microplus* and the diseases that this tick can transmit contribute to such health problems. This parasite is considered responsible for notable economic losses in livestock holdings [4], therefore its control deserves special attention.

The occurrence of ticks of the genus *Rhipicephalus*, among others, in sheep has been widely reported in Brazil and other countries [5-13]. In Brazil, specific studies have largely been restricted to the sheep industry in the northeastern region due to the traditional breeding of hair sheep that are able to withstand the local climatic conditions. In the southern region, where the weather is temperate, production of wool and fur represents additional gains because wool-producing breeds are preferred.

The occurrence of ticks, particularly *R. microplus*, in hair sheep breed species is of increasing concern due to the rapid expansion of the sheep industry into new areas traditionally used only for cattle and horses, as observed in the State of Mato Grosso do Sul.

Considering the risk of *R. microplus* infestation when sharing pastures or from neighboring pastures, the present study evaluated the ability of the hair sheep breed to maintain parasite populations after the withdrawal of the bovines as the tick source.

## Methods

### Study area and animals used

The observations were conducted in an experimental field on the Embrapa Beef Cattle farm, Campo Grande, MS, at the coordinates 20.448779 S and 54.713643 W. Temperature and rainfall data were obtained from a climatological station located at an approximate altitude of 530 meters and coordinates 20° 27’ S and 54° 37’ W. According to the Köppen & Geiger [14] classification, the area of this study lies in the transition between the Cfa humid climate, without a dry season, and the tropical humid AW climate, with a rainy season in summer and a dry season in winter.

Two paddocks were utilized, each of which had an area of 0.5 ha containing *Brachiaria decumbens*. Paddock A was infested with *Rhipicephalus microplus*, and the proximal paddock B was free of ticks. Cattle infested with *R. microplus* previously occupied paddock A for a period of four days (February 21 to February 24 of 2013). After the withdrawal of these bovines, nine pregnant Santa Inês sheep were placed in the same pasture. After 41 days, the sheep were naturally infested by ticks. This study addresses the monitoring of the natural infestation of these sheep by ticks. The observed number of sheep increased to 14 over the course of the monitoring period due to the birth of five lambs in the initial group.

### Pasture examinations for the presence of tick larvae

Between and during all tick generations, the pasture was monitored weekly by three people to observe the presence of larvae on the top of the grass leaves. For each tick generation, the pastures were monitored from the early appearance of larvae in the grass until their complete disappearance, when that generation, including the respective parasite cycle, was reported as complete.

### Sheep examinations for ticks and handling of animals between paddocks

The sheep were examined daily to register the presence of larvae and the accompanying development of ticks and determine tick detachment by counting the remaining specimens. The parasitic period comprised the time from the detection of the first larvae on the sheep to the detachment of engorged females.

When the parasitic phase was complete, the animals were removed to a nearby pasture (paddock B) to avoid possible overlapping tick generations. When larvae appeared in paddock A, the sheep were brought back and remained through the development and completion of the parasitic phase of the new tick generation. Then, the pasture transfer process was repeated. When the first larvae were again detected on the grass, the sheep were returned to paddock A for observation of the next parasitic cycle. The lambs were kept with the initial group and were subjected to the same evaluation system.

### Infection with *Babesia* and *Anaplasma*

Blood was collected from the animals for a blood smear test and for serology to identify the presence of *Babesia* and *Anaplasma*. A sample of 3 mL per animal was taken once during each parasite period, and the samples were placed into tubes containing sodium citrate (1 part citrate at 104 nmol/L plus 9 parts blood) for DNA extraction.

A 100-μl aliquot of blood was used to extract DNA using a DNAzol kit Invitrogen® (Carlsbard, US A) according to the manufacturer's recommendations. Polymerase chain reaction (PCR) for *Babesia* spp. was performed using the primers KB-16 and KB-17, while the primers, Ana-F and Ana-R were used to detect *Anaplasma* spp. [15,16]. The resulting PCR products were separated by electrophoresis on a 1.5% agarose gel with ethidium bromide staining.

## Results and discussion

Tick-infested sheep were identified on April 4, 2013; based on tick size, the ticks were estimated to be approximately six days into the parasitic cycle. Thus, there was an estimated interval of 33 days between the removal of the cattle and early infestation (February 24 to March 29, 2013). This estimate was based on the average tick incubation period of 28 days [17], determined under environmental conditions similar to those present during the development of the observed first cycle.

The second generation began after the 48th day, as measured from the fall of the last engorged female to the appearance of larvae in the pasture (April 10 to May 28, 2013). The initial nine sheep and five lambs were returned to paddock A to be infested again on June 6, 2013, and new parasitism was observed beginning on June 11, 2013. This longer incubation period may have resulted from the presence of lower temperatures, which are common in the region during autumn.

The third generation began after the 84th day, as measured from the fall of the last engorged ticks from the sheep (July 4 to September 28, 2013). The incubation period was thus even longer in the winter due to low temperatures than in the previous period. The sheep were returned to paddock A to be infested again on May 10, 2013, in the spring, and parasitism was observed beginning on October 26, 2013.

Based on the above information, we concluded that the hair sheep breed can be parasitized by *R. microplus*, and that the sheep can maintain infested areas and become infested again for at least three successive generations.

**Figure 1:**
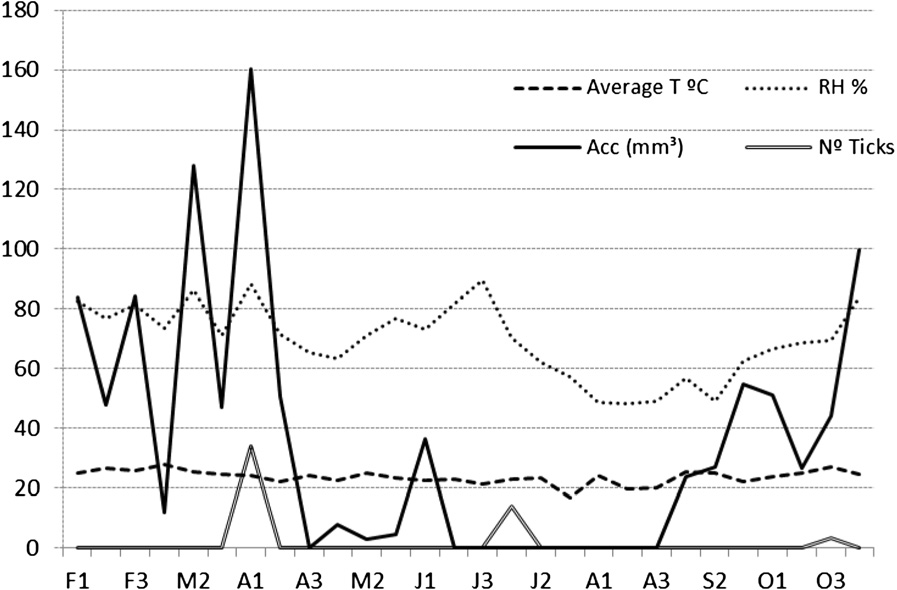
**Average number of ticks per animal resulting from the natural infestation of a Santa Ines hair sheep breed maintained in** ***Brachiaria decumbens*** **pastures without cattle for three periods of infestation.** The climatological data for Campo Grande, MS, from February to November 2013 are also shown. As an example, F1, F2, and F3 represent the ten-day intervals between observations during the month of February and so on for each indicated month. T = temperature; RH = relative humidity; Acc. = Rainfall (total for the period); and ticks = average number of individuals per animal.

The average number of ticks on the animals was recorded every ten days during each of the three observed generations, and these data are presented in Figure 1. We found that the parasitic phase in each generation lasted an average of 25 days and that the population peaks gradually diminished. While the average infestation per animal in the first generation was 34 ticks, this number decreased to an average of 12 in the second generation and 4 in the third generation.

The climatic data presented in Figure 1 show that rainfall immediately preceded the beginning of each parasitic period for the three observed generations, and a total absence of rainfall occurred from the middle of June until the end of August. The dry period of the year for the studied region extended from mid-May to September. The average temperature during the second generation was slightly lower than that during the first generation, and the prolonged incubation period and beginning of infestation that marked the beginning of the third generation were attributed to the occurrence of the lowest average temperatures in this period.

The parasitic phase of the second observed generation developed in the absence of rain. Thus, drought-related effects likely contributed to the decreased survival rate of the eggs and/or larvae, which was reflected in the low population levels observed in the third generation. From this study, we can infer that sheep play a role in the maintenance of *R. microplus,* and that ticks can be maintained for at least three generations (9 months) in the absence of cattle.

Blood smear tests did not detect presence of *Anaplasma* spp. or *Babesia* spp. However, PCR analysis of a lamb confirmed the presence of *Anaplasma* spp. PCR performed on blood from the lamb's mother did not confirm vertical transmission, suggesting that the infection was transmitted by a tick or by some other means. In a previous study of sheep in the semi-arid region of Brazil, 16-17% of the analyzed samples were positive for antibodies to *Anaplasma* spp., suggesting that species of this genus may infect small ruminants [18] and such an infection may have occurred at the study location.

## Conclusion

Santa Inês sheep are sensitive to natural infestation by the cattle tick *Rhipicephalus microplus* and are able to maintain a tick population in the pasture for at least three generations. There was a gradual decrease in the population levels of *R. microplus* over the three generations observed.
